# Genome-Wide Hypomethylation in Head and Neck Cancer Is More Pronounced in HPV-Negative Tumors and Is Associated with Genomic Instability

**DOI:** 10.1371/journal.pone.0004941

**Published:** 2009-03-18

**Authors:** Kristy L. Richards, Baili Zhang, Keith A. Baggerly, Stefano Colella, James C. Lang, David E. Schuller, Ralf Krahe

**Affiliations:** 1 Department of Genetics, University of Texas M. D. Anderson Cancer Center, Houston, Texas, United States of America; 2 Department of Bioinformatics and Computational Biology, University of Texas M. D. Anderson Cancer Center, Houston, Texas, United States of America; 3 Department of Thoracic/Head and Neck Medical Oncology, University of Texas M. D. Anderson Cancer Center, Houston, Texas, United States of America; 4 Division of Cancer Medicine, University of Texas M. D. Anderson Cancer Center, Houston, Texas, United States of America; 5 Graduate Program in Human and Molecular Genetics, University of Texas at Houston Graduate School in Biomedical Sciences, Houston, Texas, United States of America; 6 Graduate Program in Genes and Development, University of Texas at Houston Graduate School in Biomedical Sciences, Houston, Texas, United States of America; 7 Molecular Biology and Cancer Genetics Programs, Ohio State University, Comprehensive Cancer Center, Columbus, Ohio, United States of America; National Institute on Aging, United States of America

## Abstract

Loss of genome-wide methylation is a common feature of cancer, and the degree of hypomethylation has been correlated with genomic instability. Global methylation of repetitive elements possibly arose as a defense mechanism against parasitic DNA elements, including retrotransposons and viral pathogens. Given the alterations of global methylation in both viral infection and cancer, we examined genome-wide methylation levels in head and neck squamous cell carcinoma (HNSCC), a cancer causally associated with human papilloma virus (HPV). We assayed global hypomethylation levels in 26 HNSCC samples, compared with their matched normal adjacent tissue, using Pyrosequencing-based methylation assays for LINE repeats. In addition, we examined cell lines derived from a variety of solid tumors for LINE and SINE (*Alu*) repeats. The degree of LINE and *Alu* hypomethylation varied among different cancer cell lines. There was only moderate correlation between LINE and *Alu* methylation levels, with the range of variation in methylation levels being greater for the LINE elements. LINE hypomethylation was more pronounced in HPV-negative than in HPV-positive tumors. Moreover, genomic instability, as measured by genome-wide loss-of-heterozygosity (LOH) single nucleotide polymorphism (SNP) analysis, was greater in HNSCC samples with more pronounced LINE hypomethylation. Global hypomethylation was variable in HNSCC. Its correlation with both HPV status and degree of LOH as a surrogate for genomic instability may reflect alternative oncogenic pathways in HPV-positive versus HPV-negative tumors.

## Introduction

DNA methylation is an epigenetic DNA modification that occurs via the action of DNA methyltransferases on CpG dinucleotides. Methylated regions of DNA are associated with chromatin remodeling, generally occurring in areas of more condensed chromatin and decreased transcriptional activity [1], [2]. Renewed interest in this process arose after it was recognized that two types of aberrant methylation patterns are present in cancer cells [1], [3]. The first is gene-specific hyper-methylation, where CpG islands in the promoter regions of genes acquire increased methylation, generally leading to reduced expression of the downstream gene. The second is genome-wide hypo-methylation, a large percentage of which occurs in repetitive DNA elements. In malignancy, global methylation is often aberrantly reduced, whereas gene-specific methylation is often aberrantly increased. While the effects of gene-specific hypermethylation (*e.g.*, reduced expression of a gene that is important for growth control) are easily appreciated, the effects of reduced global methylation are more vague [1], [3].

It has been hypothesized that DNA methylation initially evolved as a defense mechanism against viral and other DNA pathogens as a way to silence foreign DNA sequences [4]–[6]. This is consistent with the observation that LINE and SINE (*Alu*) elements, originating from transposable elements, are heavily methylated in normal cells. Methylation of the HPV viral genome upon integration into the host genome has been reported, and changes in methylation of HPV DNA have been associated with tumorigenesis [7], [8].

Global methylation is also clinically relevant, as demonstrated by associations between clinical outcome and global methylation levels in a number of cancer types [9]–[11]. From a mechanistic standpoint, global methylation appears to be related to cancer progression, since loss of global methylation tends to become more pronounced as precancerous lesions progress [12], [13]. Furthermore, in colon cancer cells, loss of LINE methylation is inversely correlated with microsatellite instability and is directly correlated with chromosome instability [14], [15].

We hypothesized that global methylation levels in cancer, represented by levels of LINE and SINE (*Alu*) methylation, might be correlated with viral infection. We, therefore, examined LINE methylation in relation to HPV status in head and neck cancers. In contrast to cervical cancers, which are nearly all associated with HPV infection, HNSCC is virally mediated in only a subset of cases (25–30%) [16]. To determine the impact of viral infection on global methylation levels in HNSCC, we developed Pyrosequencing-based methylation assays for repetitive DNA elements and compared HPV-positive with HPV-negative cancers. Previous publications have suggested that LINE methylation is variable in HNSCC, but did not find an association between HPV DNA status and LINE methylation levels [17], [18]. Here we show that global hypomethylation is variable in HNSCC and correlates with both HPV status and genomic instability.

## Results

### LINE/SINE (*Alu*) assays are precise and reproducible, but show only moderate correlation between LINE-1 and *Alu* methylation levels

To assay global methylation levels, we adapted our Pyrosequencing-based Methylation Analysis (PMA) assay [19] to assess methylation of repetitive LINE and SINE (*Alu*) elements, similar to genome-wide methylation assays reported previously [20]. To validate the assays, we performed a series of tests: (1) mixing experiments with known amounts of methylated/unmethylated DNA; (2) methylation studies on a variety of cancer cell lines to compare LINE to SINE methylation and to survey global methylation across a broad range of malignancies; and (3) methylation of samples from different ages and genders to eliminate these factors as possible confounders of global methylation measurements.

Stepwise increments of methylated DNA were prepared by mixing universally unmethylated (U2M.L) DNA with universally methylated (UM.L) DNA in various proportions. The mixed samples were then bisulfite treated and subjected to our PMA LINE-1 (LINE) and *Alu* (SINE) assays. Linear regression analysis showed that LINE-1 and *Alu* methylation levels were very closely associated with levels predicted by input fraction of methylated DNA (*r* = 0.995, *p*-value<0.0005 for LINE-1, and *r* = 0.980, *p*-value<0.0005 for *Alu*; Figure S1). It should be noted that even when input DNA was completely methylated, the maximum global methylation percentage as measured by the PMA assay is just over 50%, not 100%. This is because individual repetitive elements have diverged in sequence over time; and CpG dinucleotides, if mutated to TpG dinucleotides, are indistinguishable from unmethylated CpGs after the bisulfite treatment. This level of background noise is taken into account by normalizing each result to the universally methylated control (*i.e.*, reporting the percent methylated reference, or PMR).

Four pools of normal DNA samples (from peripheral blood leukocytes) were generated to assess the influence of age and gender on LINE-1 and *Alu* methylation levels. Each pool (females ≤40 years old, males ≤40 years old, females >40 years old, males >40 years old) contained DNA from at least five individuals. There were no sex- or age-dependent differences between the pools in LINE-1 or *Alu* methylation levels (data not shown).

**Figure 1:**
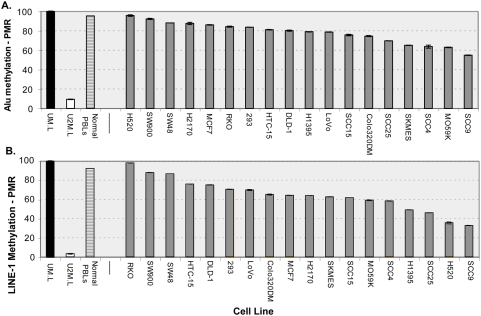
*Alu* and LINE-1 methylation in a variety of tumor cell lines. A. *Alu* methylation using the Alu-3 assay. B. LINE-1 methylation using the L1-3 assay. Results are reported as the PMR (percent methylated reference) normalized to the universally methylated reference DNA. Universally unmethylated (U2M), universally methylated (UM) controls, and normal control DNA from PBLs are also shown.

Each LINE-1 and *Alu* methylation assay was tested on a panel of 23 cancer cell lines. Different CpG sites were compared by means of three distinct LINE-1 assays and three distinct *Alu* assays, each derived from different regions of the LINE-1 and *Alu* consensus sequences, respectively (Table 1). As a control, we also tested seven normal lymphoblastoid cell lines, which showed normal levels of methylation (all >80% in our LINE-1 assays). In contrast, as expected from previous reports [12]–[14], [21], many of the tumor cell lines showed global hypomethylation, which was most pronounced in the LINE-1 assays (Figure 1). To check the consistency between LINE and SINE methylation levels (represented by our LINE-1 and *Alu* assays, respectively), we compared the degree of correlation between results from the three LINE-1 assays, between results from the three *Alu* assays, and between the various LINE-1 and *Alu* assays. Linear regression analysis showed that the individual LINE-1 assay results were highly correlated with each other, for example *r* = 0.93 for the correlation between the L1-2 and L1-3 assay results (Figure S2A). The same high degree of correlation was present between results from the *Alu* assays, for example *r* = 0.82 for the correlation between the Alu-1 and Alu-3 assay results (Figure S2B). However, LINE-1 assay results were only moderately correlated with *Alu* assay results, for example *r* = 0.33 comparing L1-1 and Alu-1; Figure S2C). A similar level of moderate correlation between LINE-1 and *Alu* assays was reported previously in neuroendocrine tumors [22].

**Table 1:** LINE-1 and *Alu* PMA Assays.

Assays	GenBank Number [Ref.]	F Primer Seq (5′–3′)	R Primer Seq (5′–3′)	Sequencing Primer (5′–3′)	Amplicon Size (bp)	No. CpG Sites
L1-1	X52235	tttattagggagtgttagatagtggg	GACGGGACACCGCTGATCGTTTA	tgggygtaggttagtgggtg (F)	117	6
			cttcccaaataaaacaatacc			
L1-2	X52235	GACGGGACACCGCTGATCGTTTA	ccctcctaaccaaatacaaaat	ccaaatacaaaatataatct (R)	196	4
		ggtattgttttatttgggaag				
L1-3	M80343	attagggagtgttagatagtggg	GACGGGACACCGCTGATCGTTTA	gygtaggttagtgtgtgtg (F)	123	5
			ccccttacrcttcccaaat			
Alu-1	[26]	GACGGGACACCGCTGATCGTTTA	ccaaactaaaatacaataa	aaactaaaatacaataac (R)	170	3
		tttttattaaaaatataaaaaattagt				
Alu-2	[26]	GACGGGACACCGCTGATCGTTTA	tcaacctcccraataactaaaa	aataactaaaattacaaac (R)	96	5
		tttttattaaaaatataaaaaattagt				
Alu-3	J00085	gagagaattgtttgaatttagga	GACGGGACACCGCTGATCGTTTA	tgaatttaggaggtgga (F)	102	4
			cactatcacccaaactaaaataca			

The sequence for the biotin-labeled universal primer (5′–3′) for the 3-primer PCR is underlined, Biotin- GGGACACCGCTGATCGTTTA . F, sequencing primer extends in the forward direction; R, sequencing primer extends in the reverse direction. r, purine (A or G); y, pyrimidine (C or T).

### LINE-1 hypomethylation of HNSCC patient samples

**Figure 2:**
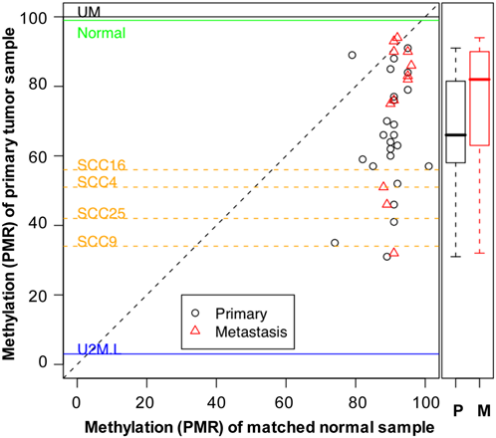
LINE-1 methylation in HNSCC tumor samples. Paired tumor and normal samples and, where available, lymph node metastases were assayed for LINE-1 methylation. PMR values are plotted using the normal sample PMR as the x-value and primary tumor (circles) or metastasis (triangle) PMR as the y-value. Horizontal lines represent PMR values for universally methylation (UM) and unmethylated (U2M) controls, normal lymphocytes (green) and for four HNSCC cell lines (yellow). Box plots on the right show the mean and distribution of primary tumors and metastases in the subset of samples where matched metastases and primary tumors were available.

Matching normal, primary tumor, and where available, lymph node metastases of head and neck patient samples (Table S1) were tested using our PMA LINE-1 assays (Figure 2). Because of the greater dynamic range of the LINE-1 assay and limited sample amounts, only LINE-1 assays were performed for the remainder of this study. In general, HNSCC primary tumors and metastatic lymph nodes were hypomethylated compared to their matching normal adjacent tissues. With the LINE1-1 assay, the mean primary tumor PMR was 65.5%, while the mean normal PMR was 90.0% (*p*-value = 6.7×10^−8^ using a paired *t*-test). However, there was quite a bit of variability in the primary tumors, ranging from 31.2% (severe hypomethylation) to 90.8% (normal). The mean PMR in lymph node metastases (73.8%; range 31.8%–93.8%) was also highly variable, even with respect to the corresponding primary tumor, sometimes lower and sometimes higher than the PMR of the primary tumor with which it is associated.

### HPV-positive tumors retain more LINE-1 methylation than HPV-negative tumors

**Figure 3:**
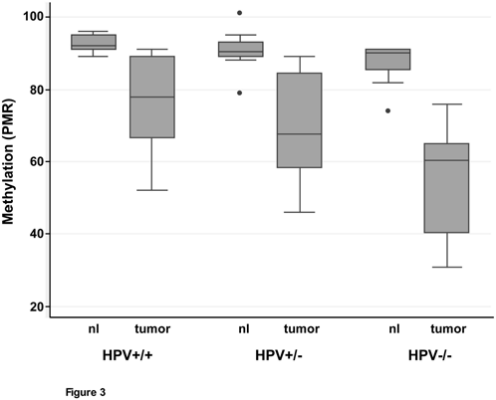
LINE-1 methylation levels are lower in HPV-negative HNSCC. Box plots of methylation levels (PMR) of HNSCC primary tumors (right in each pair) and normal adjacent tissue (left in each pair) are shown according to HPV status. +/+, HPV-positive and expressing E6 viral mRNA; +/−, HPV-positive but transcriptionally silent; and −/−, HPV negative.

Because there was so much variability in the HNSCC tumor PMR, we hypothesized that the level of global hypomethylation might vary in different subgroups of HNSCC. To explore the relationship between loss of LINE-1 hypermethylation and HPV status, we compared the mean LINE-1 methylation level of three groups that differed by their HPV status. The first group (n = 8) was HPV negative (−/−); the second group (n = 8) contained HPV DNA, yet were transcriptionally silent with respect to the E6 viral oncogene, indicating lack of expression of this oncoprotein (+/−). The third group of tumors (n = 8) was positive for HPV DNA and E6 expression (+/+). The mean methylation level of the three groups of tumors is statistically different, with a *p*-value = 0.011 for the trend (Figure 3).

We also compared global methylation levels in matched normal adjacent tissues from the same three groups. In contrast to the results for the primary tumors, there were no differences in global methylation levels in the matching normal tissues and therefore no correlation with HPV status (Figure 3). Based on a previously reported association between LINE-1 methylation and T-N-M stage [18], we looked for such an association in our sample set, but found none (*p*-value = 0.31).

### Levels of LINE-1 hypomethylation and LOH in HNSCC tumors are correlated

**Figure 4:**
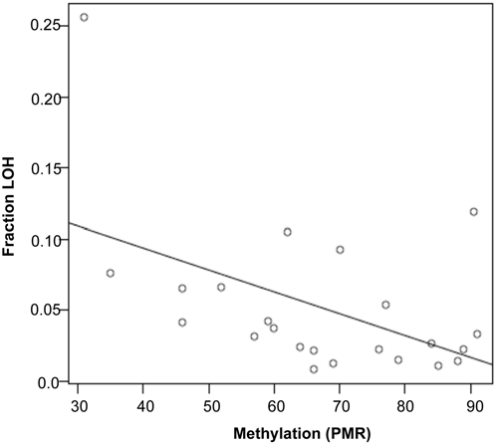
LINE-1 hypomethylation is correlated with degree of LOH in HNSCC. LINE-1 methylation is plotted as PMR, and LOH is the fraction of 10K SNP loci that show LOH relative to all informative loci. Pearson correlation coefficient is −0.494 (*p*-value = 0.01) for the correlation between the two values.

Colon cancer cell lines with more pronounced LINE hypomethylation were previously reported to have a higher degree of LOH [14], [15]. To examine whether this correlation held true in our HNSCC patient samples, we performed a global LOH analysis using 10K SNPChip data. Genotypes were compared between normal adjacent tissue and matched tumor samples; informative loci were those that were heterozygous in the normal tissue. Figure 4 shows a plot of the percentage of informative loci for each primary tumor with LOH versus the degree of LINE-1 methylation in the same specimen. We fit a linear trend to the data, which showed a Pearson correlation of −0.494, with a *p*-value = 0.017. As a check of robustness, we also calculated the Spearman (rank-based) correlation, which was also significant. The Spearman correlation coefficient for this relationship was −0.428 (*p*-value = 0.042), establishing that LOH was indeed significantly correlated with the degree of LINE hypomethylation.

## Discussion

We developed several LINE (LINE-1) and SINE (*Alu*) methylation assays with PCR primers in conserved regions of these repetitive elements. These assays utilize multiple CpG sites (3–6) to determine methylation levels and enable the simultaneous amplification of many individual LINE and SINE elements throughout the human genome as representative genomic landmarks for global methylation analysis. As expected, results from individual LINE-1 assays were very well correlated with results from other LINE-1 assays, despite measuring methylation in different regions of the LINE-1 sequence. The same is true for *Alu* assays, with high correlation between results from three different assays. However, when LINE-1 and *Alu* methylation levels were compared with each other, the correlation was more modest, only about 40%. The reasons for this lower correlation may be related simply to differences in assay sensitivity: LINE-1 assays ranged from 0–50% methylated in mixing studies, while *Alu* assays had less amplitude, ranging from 0–30% methylated, possibly secondary to higher inter-individual background noise due to sequence variation between individual *Alu* elements (Figure S1). Another possibility is that there is a functional or biological difference between the two types of repetitive DNA in their methylation maintenance. LINE repeats are more frequent in gene-poor regions of the genome, while *Alu* elements are more common in gene-rich regions [23]. Since global methylation can be measured by a variety of methods, these inter-assay differences should be considered when comparing results using different types of assays and studies.

Our results show decreases in global methylation in cell lines from a variety of cancer cell types (Figure 1), similar to previously published results for a variety of tumor types [12]–[14], [21]. It is worth noting that the cancer cell lines in general had lower levels of methylation than the HNSCC samples, perhaps reflecting a longer time to accumulate methylation loss or the clonal nature of these cell lines. However, some cell lines (e.g., RKO) had little if any loss of methylation. This implies, as for the primary HNSCC tumors, that there is variability in the underlying biology. Further research into what causes this variability could provide important information about pathways of and the role of epigenetic alterations in cancer progression.

Our results demonstrate a positive correlation between maintenance of normal LINE methylation and HPV-positivity. In addition, maintenance of normal LINE methylation was also correlated with less LOH or genome instability. If LOH is viewed as a surrogate for chromosome instability (CIN), this result is consistent with the previously reported result that colon cancers also have an association between CIN and loss of LINE hypermethylation [14], [15]. Thus, it is tempting to speculate that LINE methylation and CIN are causally related, perhaps via methylation's known association with more densely chromatin packaging, which may translate into more fidelity during chromosome segregation and therefore less LOH. However, our results and previously reported results are purely correlative; functional studies will be necessary before this causal relationship can be established.

The novel finding in our results is that HPV-positivity is correlated with maintenance of LINE methylation. A recent epidemiologic study identifying risk factors for LINE-1 hypomethylation reported no significant association between HPV DNA status and LINE-1 methylation levels [17]. However, there were methodological differences between that and our study, including the use of both HPV E6 DNA and RNA status in the assignment of HPV status, the use of a different methylation assay, and the use of methylation level as a continuous variable in the analysis. Although Furniss and colleagues did not show a direct association, they showed that outcomes varied by LINE-1 methylation levels for HPV-negative tumors [23]. Further studies are needed to clarify the association between HPV status and LINE methylation. One possible hypothesis is that in an attempt to silence the HPV virus, infected cells induce a more exuberant methylation response, harkening back to the origins of methylation as a viral defense mechanism. Although this will again require mechanistic studies or perhaps studies in additional types of virally mediated cancers (e.g., Hepatitis B and C mediated HCC compared with non-virally induced HCC, or EBV+ lymphoma vs. EBV- lymphoma) to establish causality, our results along with those of Furniss and colleagues [17] certainly provide more support for the hypothesis that HPV-positive HNSCC and HPV-negative HNSCC represent distinct biologic entities that arise via separate oncogenic pathways.

In summary, we developed several new LINE (LINE-1) and SINE (*Alu*) whole-genome methylation assays, which we applied to determine the methylation status of a variety of cancer cell lines and HNSCC primary tumor samples. We find that cancer cell lines in general have decreased methylation levels of repetitive elements. Even more variability in LINE-1 methylation was noted within HNSCC samples. Importantly, we discovered a correlation between HPV-negativity, increased genome instability, and loss of genome methylation. This correlation reinforces the concept that HPV-positive and HPV-negative cancers are biologically distinct and provides a basis for future studies to further define the biologic mechanism underlying these findings.

## Materials and Methods

### Patient samples and cell lines

Matched tumor/normal adjacent tissue samples, and when available cervical lymph node metastases from HNSCC patients were collected at Ohio State University as described previously (Table S1) [24]. Genomic DNA was extracted from the DNA-protein phase of TriZol-extracted tissues according to the manufacturer's suggestions (Invitrogen). DNA was extracted using the PureGene kit (Gentra) on cell pellets from four HNSCC cell lines (SCC-4, SCC-9, SCC-15 and SCC-25), five lung cancer cell lines (H1395, H520, H2170, SK-MES-1 and SW-900), one breast cancer cell line (MCF7), one cervical cancer cell line (HeLa), three brain cancer cell lines (U251, SK-N-AS and M059K), one uterine cancer cell line (AN3CA), one sarcoma cell line (HT1080), one kidney cancer cell line (HEK293), and six colon cancer cell lines (LoVo, SW48, HCT-15, DLD-1, COLO 320DM and RKO) according to the manufacturer's suggestions. In addition, DNA from the lymphoblastoid cell line BL1395 was used as a matching control to H1395. All cell lines are available from ATCC (Manassas, VA).

### Normal pools and methylated controls

Four pools of normal samples were generated representing different genders and ages. DNA samples were obtained from anonymous blood donors and were a gift of Dr. Michael J. Siciliano (University of Texas M. D. Anderson Cancer Center). Three of the pools (females older than 40 years of age, females age 40 or under, and males age 40 or under) were each comprised of five individuals per pool. The fourth pool (males older than 40 years of age) was comprised of six individuals. Commercially prepared universally methylated and universally unmethylated DNA (UM.C and U2M.C, respectively) were obtained from Chemicon. UM.L (universally methylated DNA ) was generated as a positive control by treating normal peripheral blood leukocyte (PBL) DNA with the CpG methylase *M.Sss*I (New England Biolabs) [25]. U2M.L (universally unmethylated DNA) was generated as a negative control by amplifying the same DNA used to generate the positive control DNA using the GenomiPhi kit (GE Healthcare) as described by the manufacturer.

### Primers and PCR conditions

PCR primers and sequencing primers were designed by using PSQ Assay Design software (Biotage) [26]. Three assays for SINE (*Alu*) elements and three assays for LINE-1 elements were designed (Table 1). PCR was performed in a 25 µl reaction containing Qiagen HotStart Taq master mix (Qiagen) using 1 µl bisulfite treated DNA (10 ng of DNA equivalents). To reduce the cost per assay, the amplification protocol was developed using a biotinylated universal primer approach. Final primer concentrations were 10 nM of the primer tailed with the universal primer, 100 nM of the untailed primer, and 90 nM of the universal biotinylated primer in each reaction [19]. The amplification was carried out at the following conditions: denaturation at 95°C for 5 min, followed by 45 cycles at 95°C for 30 sec, 45°C (SINEs) or 53°C (LINEs) for 1 min, 72°C for 45 sec, and a final extension at 72°C for 7 min [19].

### PyroMethA (PMA) and methylation assessment

Bisulfite conversion of genomic DNA was done as reported previously [19]. Briefly, 0.5–1.0 µg of genomic DNA was treated using the CpGenome DNA modification kit (Chemicon), including DNA sulfonation, deamination, desalting, desulfonation and recovery. Bisulfite-treated DNA was stored at −20°C until use. PMA is a Pyrosequencing-based technology that can analyze CpG methylation at multiple sites in a single assay. After a PCR amplification using bisulfite treated DNA, Pyrosequencing was carried out using the PSQ96HS system (Biotage) according to the manufacturer's protocol including single strand binding protein (PyroGold reagents). The results were analyzed using Q-CpG software (Biotage), which calculates the methylation percentage (^m^C/(^m^C+C)) for each CpG site, allowing quantitative comparisons. The methylation index (called MI) was calculated as the average value of ^m^C/(^m^C+C) for all examined CpG sites in the assay. In general, there was very good agreement in methylation levels among individual CpG sites in the same assay. Bisulfite-treated UM.L was used as the universally methylated reference. PMA data for these global methylation assays are reported as a percentage of methylated reference (PMR) value, normalizing the MI of each sample to the MI of the universally methylated reference (UM.L) DNA [25].

Commercial universally methylated DNA (UM.C) and universally unmethylated DNA (U2M.C) (Chemicon) were also used to perform this analysis, and the result was linear (*r* = 0.983, *p*-value<0.0005 for LINE-1; Figure S1). However, commercially available U2M was not completely unmethylated, since a pure U2M.C sample had residual methylation of approximately 26%, different from U2M.L prepared in our lab, which had the expected 0% methylation. We therefore used our own universally unmethylated DNA (U2M.L) for all further studies.

### Detection of HPV16 E6 DNA and E6 RNA to determine HPV status

Quantitative real-time PCR was performed to detect either HPV16 E6 DNA or E6 cDNA using the same primer/probe set for both. The primers were designed using the HPV16 serotype, which accounts for ≥90% of all HPV-positive HNSCC cases [27]. To control for possible genomic DNA contamination in the cDNA, amplifications of cDNA from DNAse-treated RNA that had been prepared using the SuperScript First Strand Synthesis System (Invitrogen) both with and without the addition of reverse transcriptase were compared. PCR was performed in a 25 µl reaction containing iQ supermix master mix (Biorad) using 25 ng of genomic DNA or 5 µl of cDNA. Forward primer (5′-CTGCAATGTTTCAGGACCCA-3′) and reverse primer (5′-TCATGTATAGTTGTTTGCAGCTCTGT-3′) were added to a final concentration of 200 nM each. The Texas Red labeled real-time probe (5′-TR-AGGAGCGACCCAGAAAGTTACCACAGTT-3′) was added to a final concentration of 320 nM. Fluorescein was added to each reaction at a final concentration of 10 pM. Each reaction was performed in triplicate. The amplification was carried out at the following conditions: denaturation at 95°C for 8.5 min, followed by 50 cycles at 95°C for 15 sec, 60°C for 1 min and analyzed in real time using an iCycler PCR machine and software (Biorad). Samples that did not amplify were scored as negative, and all samples were grouped into 3 categories based on these results: (+/+), positive for both E6 DNA and E6 RNA; (+/−), positive for E6 DNA, but transcriptionally silent; and (−/−), negative for E6 DNA and RNA. HPV status was successfully determined for 24 of the 26 HNSCC samples, and these were used for subsequent analyses utilizing HPV status.

### 10K SNPChip LOH analysis

HNSCC genomic DNAs were extracted using the standard TriZol-extraction protocol; they were further purified by ethanol precipitation before and after whole genome amplification using the GenomiPhi kit (GE Healthcare). The Affymetrix 10K *Xba*131 array contains approximately 11,500 SNPs with an average spacing of 210 kb. Standard Affymetrix protocols were followed for these assays. Briefly, about 250 ng genomic DNA was digested with *Xba*I and then ligated to adaptors. Next, one-primer amplification was carried on by using the GeneAmp PCR System 9700 (Applied Biosystems). After purification with Qiagen MinElute 96 UF, a total of about 20 µg of PCR product was fragmented and labeled with biotin. Hybridization was performed in the Affymetrix GeneChip Hybridization Oven at 48°C for 16–18 hours. Arrays were washed and stained with the Affymetrix GeneChip Fludics Station 400 and were scanned with the Affymetrix GeneArray 2500 Scanner. Image processing was performed with GCOS 1.0 software and genotypes were generated with GTYPE 2.0 or higher software.

### Statistical analysis

In order to assess the association between levels of methylation and levels of HPV load (a similar analysis was also used to assess the association between levels of methylation and T-N-M stage), we proceeded as follows. All sample methylation values were converted to ranks. One missing value for the LINE1-1 assay, for sample P2, was imputed using the P2 value from the LINE1-3 (correlation between the LINE1-1 and LINE1-3 assays is 0.98). These ranks were then scaled (weighted) by HPV load, with weights of 0, 1 and 2 for −/−, +/−, and +/+, respectively. The final association “score” was the sum of these weighted ranks. The null distribution for this score was assessed by simulations in which we repeatedly allocated samples to HPV groups at random. We ran one million simulations, and defined our *p*-value as two times the proportion of cases in which the simulation score was as large or larger than the one we actually saw. Our simulated *p*-value was 0.011.

## Supporting Information

Table S1 Clinical and molecular features of HNSCC samples.
(0.12 MB DOC)

Figure S1 Dynamic range of PMA LINE-1 and Alu Assays. Mixing experiments were performed to determine PMA methylation levels measured with varying proportions of universally methylated and unmethylated DNA. UM.C and U2M.C represent commercially available (Chemicon) universally methylated and unmethylated DNAs, respectively. UM.L and U2M.L were generated from the same DNA by in vitro modification in our laboratory.
(0.10 MB TIF)

Figure S2 Correlation analysis of SINE and LINE global methylation assays. A variety of tumor and normal cell line DNAs were assayed with all six of our LINE and SINE assays (three LINE-1 assays and three Alu assays). A. LINE-1 assays were highly correlated with each other. B. Alu assays were highly correlated with each other. C. However, LINE-1 and Alu assays were only moderately correlated. Two representative plots are shown for each series of comparisons.
(0.19 MB TIF)
