# Supplementary material for: Genome-Wide Hypomethylation in Head and Neck Cancer Is More Pronounced in HPV-Negative Tumors and Is Associated with Genomic Instability
Source: PLoS One. 2009 Mar 18;4(3):e4941. doi: 10.1371/journal.pone.0004941 (PMC2654169; doi:10.1371/journal.pone.0004941)
Supplement: Table S1 — Clinical and molecular features of HNSCC samples. (0.12 MB DOC) [file pone.0004941.s001.doc]

| **Patient**  **No.** | **PMR-nl** | **PMR-prim** | **PMR-met** | **HPV Status** | **#LOH** | **#AB** | **#LOH/#AB** | **Primary Tumor Site** | **Sex** | **Age** | **Tc** | **Nc** | **Mc** | **Stage** |
| --- | --- | --- | --- | --- | --- | --- | --- | --- | --- | --- | --- | --- | --- | --- |
| 2 | 0.96 | 0.91 | NA | +/+ | 211 | 1776 | 0.119 | Oropharynx/tonsil | M | 47 | 4 | 2 | 0 | IV |
| 5 | 0.91 | 0.66 | 0.93 | -/- | 21 | 2532 | 0.008 | Larynx/subglottis | M | 76 | 4 | 2 | 0 | IV |
| 6 | 0.91 | 0.41 | 0.32 | NA | NA | NA | NA | Pharynx | M | 50 | NA | NA | NA | NA |
| 7 | 0.89 | 0.31 | 0.46 | -/- | 557 | 2175 | 0.256 | Larynx/supraglottis | M | 48 | 2 | 3 | 0 | IV |
| 8 | 0.95 | 0.91 | 0.9 | +/+ | 81 | 2446 | 0.033 | Oral cavity/tongue | M | 65 | 3 | 2 | 0 | IV |
| 9 | 0.95 | 0.84 | 0.83 | +/- | 56 | 2105 | 0.027 | Oral cavity/tongue | M | 45 | 3 | 1 | 0 | III |
| 10 | 0.91 | 0.88 | 0.9 | +/+ | 38 | 2674 | 0.014 | Oropharynx/tonsil | M | 54 | 1 | 2 | 0 | IV |
| 11 | 0.92 | 0.52 | 0.94 | +/+ | 187 | 2831 | 0.066 | Larynx/glottis | M | 64 | 4 | 1 | 0 | IV |
| 12 | 0.95 | 0.79 | 0.82 | +/+ | 39 | 2582 | 0.015 | Oropharynx/BOT | M | 53 | 3 | 1 | 0 | III |
| 13 | 0.92 | 0.63 | NA | +/+ | NA | NA | NA | Hypopharynx/pyriform sinus | M | 64 | 3 | 2 | 0 | IV |
| 14 | 0.91 | 0.69 | NA | +/- | 27 | 2143 | 0.013 | Oropharynx/BOT | M | 49 | 4 | 0 | 0 | IV |
| 15 | 0.88 | 0.66 | 0.51 | +/- | 57 | 2595 | 0.022 | Larynx/supraglottis | F | 54 | 2 | 1 | 0 | III |
| 16 | 0.9 | 0.64 | 0.75 | -/- | 54 | 2237 | 0.024 | Oral cavity/tongue | F | 78 | 3 | 2 | 0 | IV |
| 17 | 0.91 | 0.77 | 0.76 | +/+ | 114 | 2105 | 0.054 | Oropharynx/tonsil | M | 47 | 2 | 2 | 0 | IV |
| 18 | 1.01 | 0.57 | NA | +/- | 66 | 2105 | 0.031 | Larynx/supra | M | 56 | 3 | 0 | 0 | III |
| 19 | 0.79 | 0.89 | NA | +/- | 59 | 2635 | 0.022 | Larynx/glottis | M | 54 | 4 | 0 | 0 | IV |
| 20 | 0.82 | 0.59 | NA | -/- | 109 | 2582 | 0.042 | Larynx/glottis | M | 63 | 3 | 0 | 0 | III |
| 21 | 0.85 | 0.57 | NA | NA | NA | NA | NA | Larynx/glottis | M | 59 | 2 | 0 | 0 | II |
| 22 | 0.89 | 0.7 | NA | +/+ | 204 | 2200 | 0.093 | Hypopharynx/pyriformis | M | 56 | 2 | 3 | 0 | IV |
| 23 | 0.91 | 0.46 | NA | +/- | 96 | 2289 | 0.042 | Hypopharynx/pyriformis | M | 61 | 3 | 2 | 0 | IV |
| 24 | 0.74 | 0.35 | NA | -/- | 161 | 2109 | 0.076 | Hypopharynx/cricoid | F | 62 | 3 | 1 | 0 | III |
| 27 | 0.9 | 0.85 | NA | +/- | 27 | 2418 | 0.011 | Oral cavity/tongue | M | 67 | 2 | 0 | 0 | II |
| 28 | 0.9 | 0.6 | NA | +/- | 94 | 2486 | 0.038 | Oropharynx/palate | M | 69 | 1 | 0 | 0 | I |
| 29 | 0.91 | 0.46 | NA | -/- | 141 | 2151 | 0.066 | Oropharynx/tonsil | F | 70 | 2 | 1 | 0 | III |
| 30 | 0.9 | 0.62 | NA | -/- | 244 | 2315 | 0.105 | Oral cavity/tongue | M | 30 | 3 | 0 | 0 | III |
| 31 | 0.91 | 0.76 | NA | -/- | 59 | 2595 | 0.023 | Oropharynx/BOT | M | 51 | 4 | 0 | 0 | IV |

**Table S1.** Clinical and molecular features of HNSCC samples.

PMR-nl, PMR of normal adjacent tissue; PMR-prim, PMR of primary tumor; PMR-met, PMR of lymph node metastasis. HPV status: +/+, positive at both DNA level and for E6 transcriptional level; +/-, positive at the DNA level but transcriptionally silent for E6; -/-, negative. #LOH, number of 10K SNPs that show loss of heterozygosity; #AB, number of 10K SNPs that are heterozygous in normal adjacent tissue. Tc/Nc/Mc, clinical T-N-M stage.
