# Supplementary figures and images for: Genome-Wide Hypomethylation in Head and Neck Cancer Is More Pronounced in HPV-Negative Tumors and Is Associated with Genomic Instability
Source: PLoS One. 2009 Mar 18;4(3):e4941. doi: 10.1371/journal.pone.0004941 (PMC2654169; doi:10.1371/journal.pone.0004941)

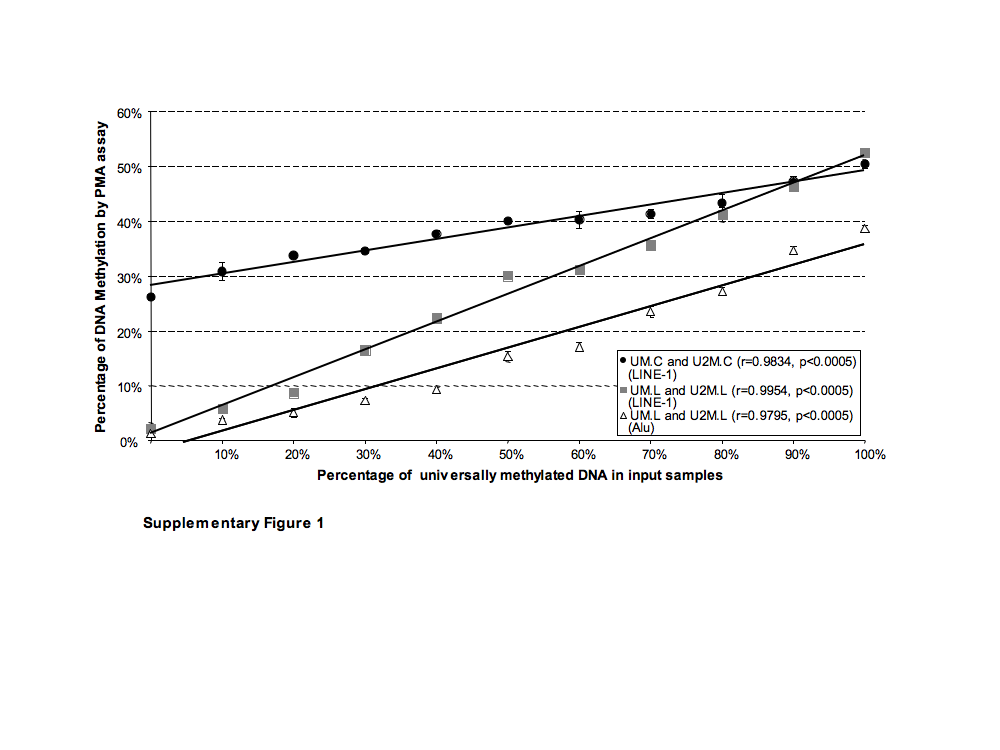

Supplement: Figure S1 — Dynamic range of PMA LINE-1 and Alu Assays. Mixing experiments were performed to determine PMA methylation levels measured with varying proportions of universally methylated and unmethylated DNA. UM.C and U2M.C represent commercially available (Chemicon) universally methylated and unmethylated DNAs, respectively. UM.L and U2M.L were generated from the same DNA by in vitro modification in our laboratory. (0.10 MB TIF) [file pone.0004941.s002.tif]

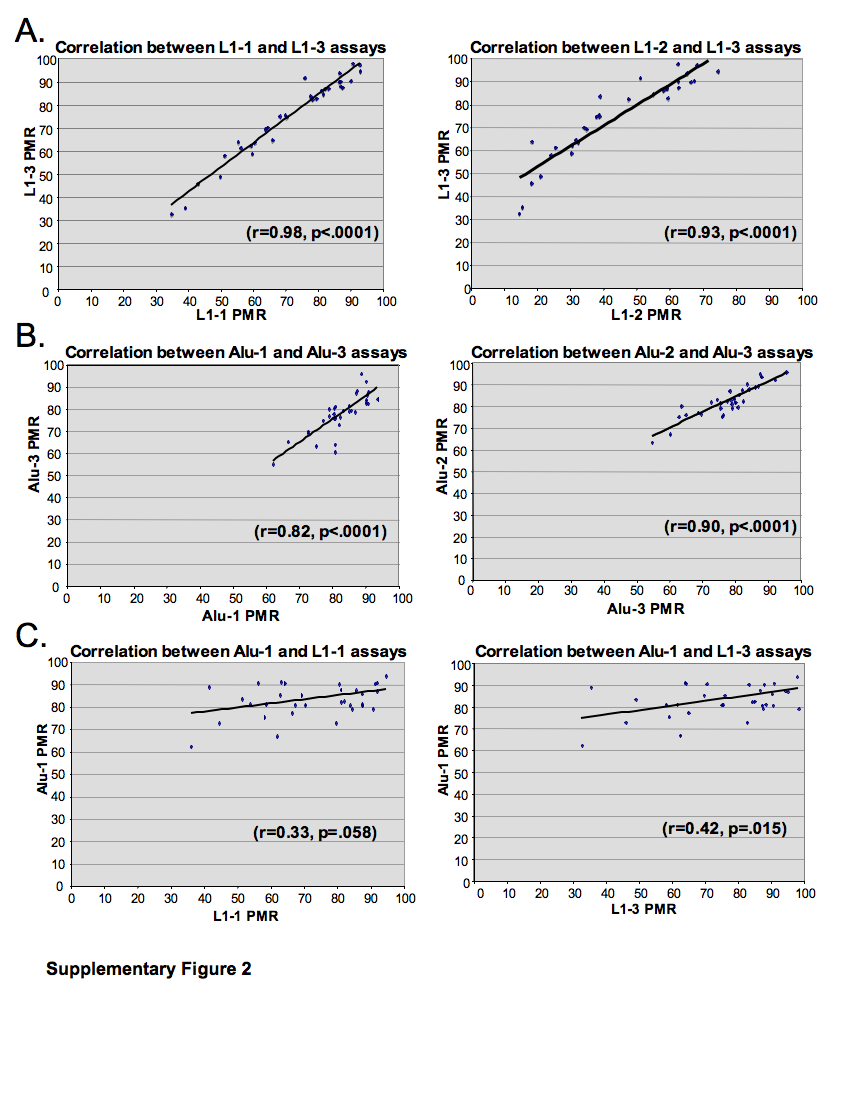

Supplement: Figure S2 — Correlation analysis of SINE and LINE global methylation assays. A variety of tumor and normal cell line DNAs were assayed with all six of our LINE and SINE assays (three LINE-1 assays and three Alu assays). A. LINE-1 assays were highly correlated with each other. B. Alu assays were highly correlated with each other. C. However, LINE-1 and Alu assays were only moderately correlated. Two representative plots are shown for each series of comparisons. (0.19 MB TIF) [file pone.0004941.s003.tif]
